# Pre-Clinical Development of BCG.HIVA^CAT^, an Antibiotic-Free Selection Strain, for HIV-TB Pediatric Vaccine Vectored by Lysine Auxotroph of BCG

**DOI:** 10.1371/journal.pone.0042559

**Published:** 2012-08-21

**Authors:** Narcís Saubi, Alice Mbewe-Mvula, Ester Gea-Mallorqui, Maximillian Rosario, Josep Maria Gatell, Tomáš Hanke, Joan Joseph

**Affiliations:** 1 AIDS Research Group, Hospital Clinic/IDIBAPS-HIVACAT, School of Medicine, University of Barcelona, Barcelona, Spain; 2 The Jenner Institute, University of Oxford, Oxford, United Kingdom; 3 MRC Human Immunology Unit, Weatherall Institute of Molecular Medicine, University of Oxford, Oxford, United Kingdom; University of Cape Town, South Africa

## Abstract

In the past, we proposed to develop a heterologous recombinant BCG prime-recombinant modified vaccinia virus Ankara (MVA) boost dual pediatric vaccine platform against transmission of breast milk HIV-1 and *Mycobacterium tuberculosis* (*Mtb*). In this study, we assembled an *E. coli*-mycobacterial shuttle plasmid pJH222.HIVA^CAT^ expressing HIV-1 clade A immunogen HIVA. This shuttle vector employs an antibiotic resistance-free mechanism based on Operator-Repressor Titration (ORT) system for plasmid selection and maintenance in *E. coli* and lysine complementation in mycobacteria. This shuttle plasmid was electroporated into parental lysine auxotroph (safer) strain of BCG to generate vaccine BCG.HIVA^CAT^. All procedures complied with Good Laboratory Practices (GLPs). We demonstrated that the episomal plasmid pJH222.HIVA^CAT^ was stable *in vivo* over a 20-week period, and genetically and phenotypically characterized the BCG.HIVA^CAT^ vaccine strain. The BCG.HIVA^CAT^ vaccine in combination with MVA.HIVA induced HIV-1- and *Mtb*-specific interferon γ-producing T-cell responses in newborn and adult BALB/c mice. On the other hand, when adult mice were primed with BCG.HIVA^CAT^ and boosted with MVA.HIVA.85A, HIV-1-specific CD8^+^ T-cells producing IFN-γ, TNF-α, IL-2 and CD107a were induced. To assess the biosafety profile of BCG.HIVA^CAT^-MVA.HIVA regimen, body mass loss of newborn mice was monitored regularly throughout the vaccination experiment and no difference was observed between the vaccinated and naïve groups of animals. Thus, we demonstrated T-cell immunogenicity of a novel, safer, GLP-compatible BCG-vectored vaccine using prototype immunogen HIVA. Second generation immunogens derived from HIV-1 as well as other major pediatric pathogens can be constructed in a similar fashion to prime protective responses soon after birth.

## Introduction

According to the last UNAIDS World AIDS Day Report 2011, at the end of 2010, an estimated 34 million people were living with HIV worldwide and 2.7 million individuals became newly infected with the virus in 2010. The number of people dying of AIDS-related causes was 1.8 million in 2010, and it is estimated that more than 16 million children have been orphaned by AIDS. Sub-Saharan Africa accounted for 70% of new HIV infections in 2010, and even though it is encouraging that 6.6 million people are currently receiving treatment in resource-poor settings, ensuring universal access to antiretrovirals still represents an enormous challenge [1]. Without access to drugs, rates of HIV-1 Mother-to-child transmission (MTCT) are 15–30% in non breastfeeding populations. Breastfeeding by an infected mother adds an additional 5–20% risk leading to an overall transmission rate of 20–45% as shown in some African and Asian settings [2]. Development of effective and safe neonatal and/or adult vaccines is the best solution to prevent infection or reduce the severity of HIV-related diseases. Infection with *Mycobacterium tuberculosis* (*Mtb*) kills about 2 million people each year and goes hand-in-hand with HIV-1. *Mycobacterium bovis* bacillus Calmette–Guérin (BCG) is the only licensed vaccine and protects significantly against childhood and milliary tuberculosis. Globally, 80% of children are vaccinated with BCG, the majority of them at birth. Thus, the development of a combined vaccine, which would protect neonates against tuberculosis and MTCT of HIV-1 through breastfeeding, is a logical effort in the fight against these two major global killers.

Only two candidate vaccines designed to protect against breast milk HIV transmission have been studied in human infants (HIV-1 gp120 recombinant subunit and live-attenuated recombinant canarypox ALVAC vaccines) [3]–[5]. Therefore, there is an urgent need for a neonatal immunogen that generates HIV-specific immunity more rapidly. Recombinant BCG (rBCG) has been developed as a candidate neonatal vaccine vector against pertussis [6], measles [7], respiratory syncytial virus (RSV) [8] and breast milk HIV transmission [9], [10]. BCG as a vaccine vector has a number of attractive features. BCG has a proven record of safety as a vaccine against tuberculosis from its use in over two billion individuals [11]. However, BCG has now been questioned for safety, especially in HIV-endemic regions where both HIV and TB are highly endemic. Currently, HIV infection in infants is now a full contraindication to BCG vaccination [12]. Nevertheless, the BCG Working Group of the International Union against Tuberculosis and Lung recommended that current universal BCG immunisation of infants continue in countries highly endemic for TB until they have all programmes in place for implementing selective deferral of HIV-exposed infants [13]. BCG infects and colonizes macrophages and dendritic cells, where it can survive and replicate for a long period of time. Through its persistence and potent adjuvantation by its cell wall components, it can induce long-lasting humoral and cellular immune responses. BCG can be given at or any time after birth, and is not affected by maternal antibodies [14], [15]. Manufacturing of BCG-based vaccines is inexpensive. Finally, BCG is one of the most heat-stable vaccines in current use [16].

There is strong evidence in favour of a role for HIV-1 specific T-cell responses in the control of HIV-1 replication [17], [18]. One promising approach for T-cell induction is *Mycobacterium bovis* BCG as a bacterial live recombinant vaccine vehicle. Specific humoral and cellular immune responses against HIV-1 have been detected after immunization of mice with rBCG expressing HIV-1 antigens [19]–[21]. For a number of years, we have been working on rBCG based HIV-1 vaccine development with the aim to induce protective cell-mediated responses. Our starting platform was based on a heterologous rBCG prime and recombinant modified vaccinia virus Ankara (MVA) boost regimen delivering a common immunogen called HIVA, which is derived from consensus Gag protein of HIV-1 clade A, prevalent in Central and Eastern Africa, and a string of CD8^+^ T-cell epitopes [22]. BCG.HIVA^222^ carrying an episomal plasmid expressing HIVA was shown to be stable and to induce durable, oligofunctional HIV-1-specific CD4^+^ and CD8^+^ T-cell responses in BALB/c mice. Furthermore, when the BCG.HIVA^222^ vaccine was used in a prime-boost regimen with heterologous vectors, HIV-1-specific responses provided protection against surrogate virus challenge expressing HIVA, and was also as efficient in protecting against aerosol challenge with *Mtb* as the BCG 1173 P2 vaccine Pasteur strain. The BCG.HIVA^222^ vaccine candidate was vectored by a lysine auxotroph of BCG Pasteur strain that carried an *E. coli*-mycobacterial shuttle plasmid pJH222.HIVA with a lysine A complementing gene and a weak promoter to drive HIVA gene expression [10]. This design increases the plasmid stability *in vivo* and prevents heterologous gene expression disruption by genetic rearrangement [23]. We also evaluated the influence of BALB/c mice age and immunization routes on induction of HIV-1 and *Mtb*-specific immune responses. Administration of BCG.HIVA^222^ to newborn mice was safe and primed HIV-1-specific immune responses boosted by subsequent MVA.HIVA administration [24]. Also, MVA.HIVA.85A, a dual AIDS and tuberculosis vaccine, was designed to boost both the *Mtb* and HIV-1-specific immune responses primed by BCG.HIVA^222^ [25].

In this study, we constructed a novel HIVA-expressing *E. coli*-mycobacterial shuttle plasmid pJH222.HIVA^CAT^ by using an antibiotic-free plasmid selection system based on Operator-Repressor Titration (ORT) system in *E. coli* and lysine complementation in mycobacteria. This plasmid DNA was electroporated into parental lysine auxotroph of BCG to generate vaccine BCG.HIVA^CAT^. The genetic and phenotypic characterization of antibiotic markerless BCG.HIVA^CAT^ strain was performed. The presence of HIVA gene sequence and protein expression by the recombinant mycobacterium were confirmed, its safety was evaluated by monitoring the body mass gain and the induction of HIV-1 and *Mtb*-specific immune responses was demonstrated in both newborn and adult BALB/c mice after BCG.HIVA^CAT^ prime and MVA.HIVA or MVA.HIVA.85A boost. The BCG.HIVA^CAT^ strain was developed in GLP-compatible conditions, properly characterized, stable *in vivo*, induced specific HIV-1 and *Mtb* immune responses in newborn and adult mice and was well tolerated in newborn mice. In addition, the compatibility with GLP requirements is relevant for progressing this novel vaccine into clinical evaluation.

## Results

### Construction of the BCG.HIVA^CAT^ vaccine strain

**Figure 1:**
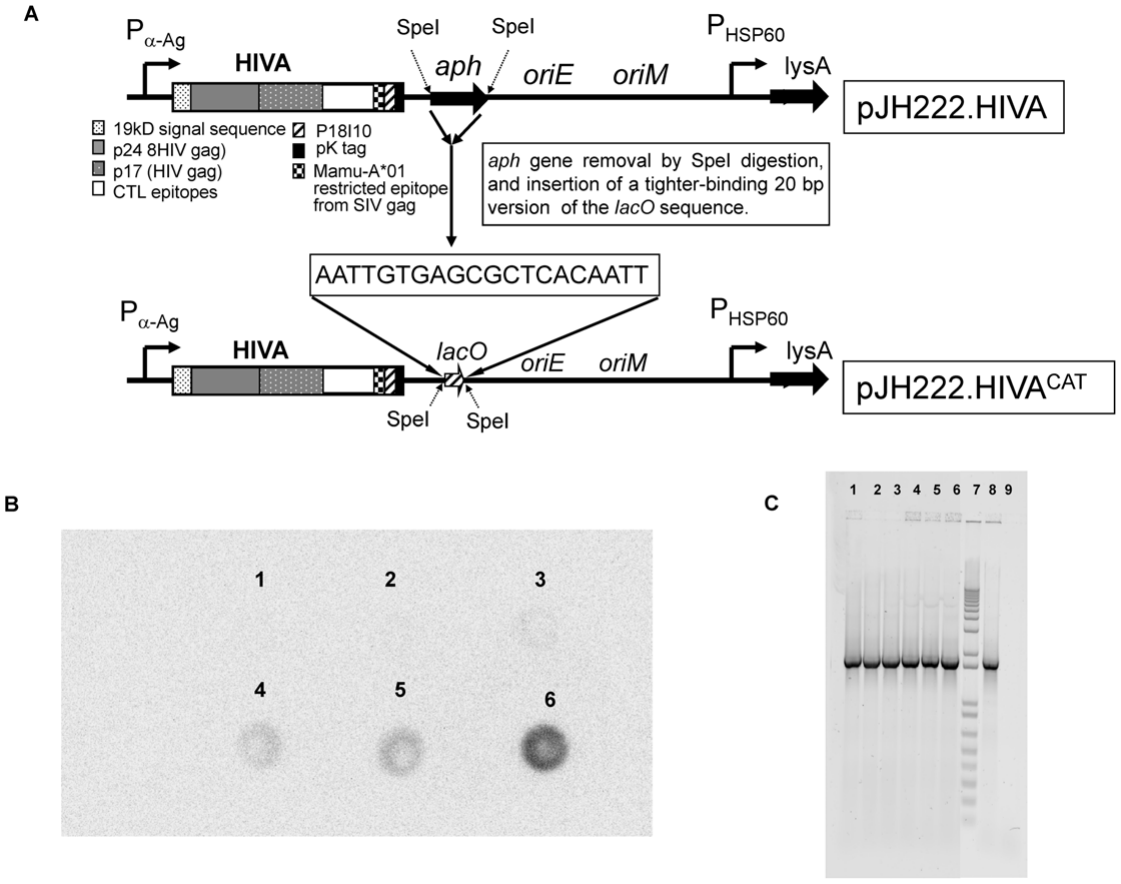
Construction of the BCG.HIVA^CAT^ vaccine strain. (**A**) A synthetic GC-rich HIVA gene was fused to the region encoding the 19-kDa lipoprotein signal sequence and inserted into the episomal pJH222 *E. coli*-mycobacterium shuttle plasmid. This plasmid contains kanamycin resistance (*aph*) and complementing *lysA* genes and an *E. coli* origin of replication (oriE). In addition, pJH222 contained the mycobacterial origin of replication (oriM). The BALB/c mouse T-cell and MAb Pk epitopes used in this work are depicted. P α-Ag, *M. tuberculosis* α-antigen promoter; P*HSP60*, heat shock protein 60 gene promoter. The *aph* gene was removed by SpeI digestion and the *lacO* sequence was inserted and transformed into *E. coli* DH1*lacdapD* strain. (**B**) Immunodot of BCG.HIVA^CAT^ lysates. Dot 1: BCG wild type (negative control); Dot 2, 3, 4 and 5: clone 3, clone 7, clone 9 and clone 10 of BCG.HIVA^CAT^; Dot 6: BCG.HIVA^222^ (positive control). HIVA peptide was detected using the anti-Pk MAb followed by horseradish peroxidase-Goat-anti-Mouse and enhanced chemiluminescence (ECL) detection. (**C**) *In vivo* plasmid stability of BCG.HIVA^CAT^ harboring pJH222.HIVA^CAT^. Mice were injected s.c. with 10^5^ cfu of BCG.HIVA^CAT^ and boosted i.m. with 10^6^ pfu of MVA.HIVA, spleens were homogenized 20 weeks after BCG inoculation and the recovered rBCG colonies were tested for the presence of the HIVA DNA coding sequence by PCR. Lanes 1 to 6: Six rBCG colonies were recovered in the non-lysine supplemented plate; lane 7: Molecular weight marker; lane 8: Plasmid DNA positive control; lane 9: Distilled water (negative control).

HIVA immunogen consists of consensus HIV-1 clade A Gag p24/p17 domains coupled to a string of CD8^+^ T-cell epitopes and monoclonal antibody (mAb) tag Pk [22]. The HIVA gene was synthesized utilizing humanized GC-rich codons, which are similar to those used by mycobacteria [26], [27]. To facilitate the pre-clinical development of candidate vaccines, the HIVA immunogen contains an immunodominant H-2D^d^-murine restricted epitope P18-I10 [28]. The HIVA open-reading frame was fused at its 5′ end to nucleotides coding for the 19-kDa lipoprotein signal sequence, which facilitates the antigen secretion and fusion of foreign antigens to mycobacterial surface lipoproteins, enhancing the foreign protein immunogenicity [29]. The chimeric 19-kDa signal sequence-HIVA gene was expressed from *E. coli-*mycobacterial shuttle plasmid pJH222 under the control of the *Mtb* α-antigen promoter (Figure 1A). Plasmid DNA pJH222 is a replicative (multicopy, extrachromosomal) vector that contains a DNA cassette encoding kanamycin resistance (Tn903-derived *aph* gene), an *E. coli* origin of replication (*oriE*) and a mycobacterial plasmid DNA origin of replication (*oriM*). It also contains the wild-type lysine A-complementing gene for the vector maintenance (*lysA5*) in the BCG auxotroph [10]. The kanamycin resistance gene was removed from pJH222.HIVA vector by using the Operator-Repressor Titration (ORT) system developed by Cobra Biologics (UK). Such system enables the selection and maintenance of plasmids that are free from expressed selectable marker genes and require only the short, non-expressed *lac* operator for selection and maintenance [30]. The principle *Escherichia coli* ORT strain, DH1*lacdapD* [31], has been used to produce several important DNA vaccine candidates such as the HIV-1 vaccine pTHr.HIVA [32]. In this work the kanamycin resistance gene was replaced with a *lac* operator sequence and the resulting plasmid, pJH222.HIVA^CAT^ was transformed into the *E.coli* DH1*lacdapD* strain (Figure 1A). When the non-expressed *lac* operator sequence was inserted into multicopy plasmid and introduced into the cell, the binding of the repressor protein to the plasmid-borne operator derepresses the chromosomal operator and allows *dapD* expression and cell growth [32]. The recombinant pJH222.HIVA containing the ORT selection system, here designated as pJH222.HIVA^CAT^, was transformed into lysine auxotroph of BCG host strain Pasteur ΔlysA5::res [33]. The selection of positive BCG.HIVA^CAT^ colonies was made by growing the rBCG cells on Middlebrook agar 7H10 medium with no supplementation of lysine. Expression of the full-size chimeric 19-kDa signal sequence-HIVA protein was confirmed by immunodot of whole transformed mycobacterial cell lysates using anti-Pk mAb. As shown in Figure 1B, the highest level of HIVA protein expression was detected after blotting the BCG culture from clone number 10 and was selected for further molecular characterization, immunogenicity and safety testing in mice. On the other hand, the BCG.HIVA^CAT^ clone10 culture was preserved by using the seed-lot system. A Master Seed stock, and derivative Working Stock, which we used also as a Vaccine stock was prepared. Growth of the transformed mycobacteria and the *in vivo* stability of pJH222.HIVA^CAT^ episomal plasmid were established by the recovery of BCG.HIVA^CAT^ colonies from the spleens of BALB/c mice 20 weeks after immunization. Six out of six recovered rBCG colonies were positive for HIVA DNA coding sequence by PCR (Figure 1C).

### Genetic characterization of the BCG.HIVA^CAT^

**Figure 2:**
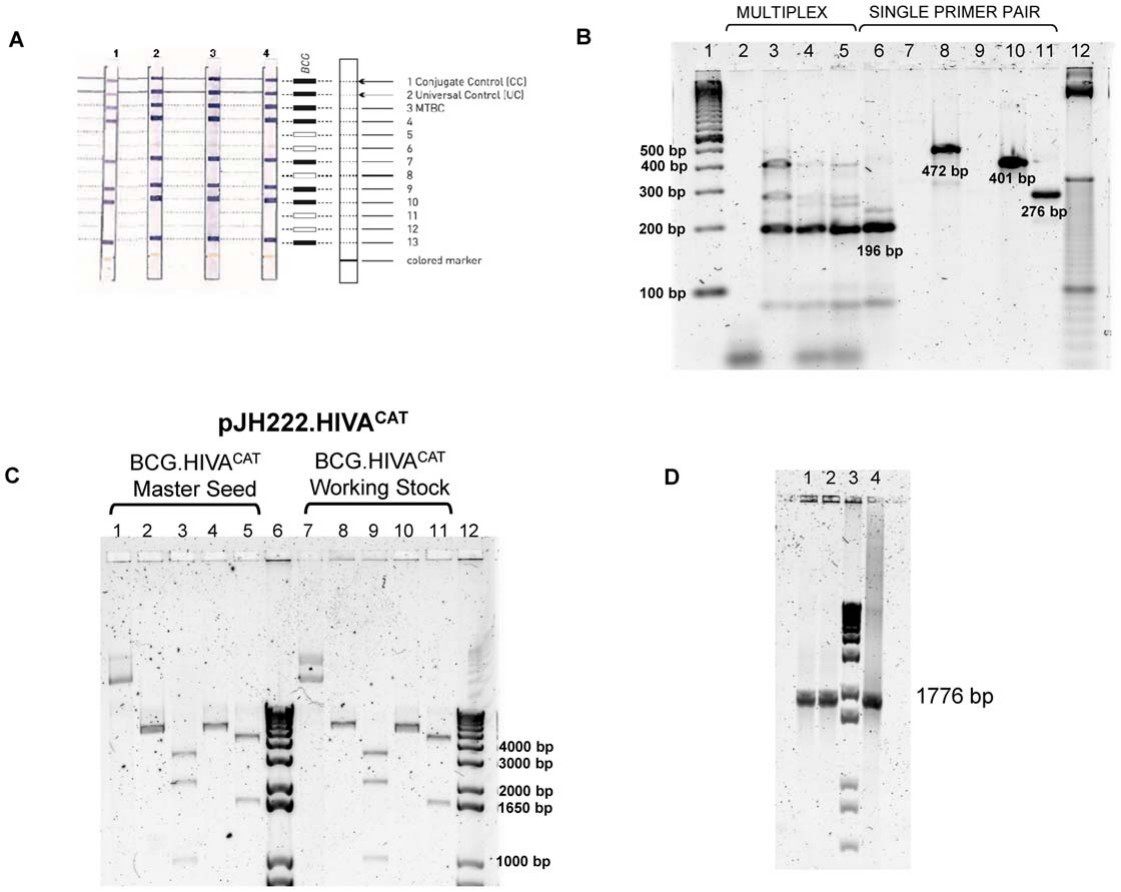
Genetic characterization of the BCG.HIVA^CAT^. GenoType MTBC assay and Multiplex PCR assay. (**A**) The BCG.HIVA^CAT^ strain identification results representative of all of the patterns obtained with the GenoType MTBC assay. The positions of the oligonucleotides, the marker line and the BCG hybridization pattern are shown on the right. The specificity and targeted genes of the lines are as follows: 1, conjugate control; 2, amplification control (23S rRNA); 3, MTBC specific (23S rRNA); 4 to 12, discriminative for the MTBC species (*gyrB*); 13, *M. bovis* BCG (RD1). The samples analyzed were: Strip 1: BCG Connaught; Strip 2: BCG.HIVA^CAT^; Strip 3: BCG.HIVA^222^; Strip 4: BCG wild type. All four strains presented the same hybridization pattern corresponding to *M. bovis* BCG. (**B**) The BCG.HIVA^CAT^ Pasteur substrain identification by multiplex PCR assay. Lane 1 and 12: molecular weight marker; lane 2: negative control; lane 3, 6–11: BCG.HIVA^CAT^(clone10); lane 4: BCG Danish strain (using 1 µl of template); lane 5: BCG Danish strain (using 4 µl template); The samples were analyzed by multiplex primer assay or single primer pair assay. Lane 2–5: multiplex primers; lane 6: ET1-3 primers; lane 7: RD2 primers; lane 8: RD8 primers; lane 9: RD14 primers; lane 10: RD16 primers; lane 11: C3–C5 primers. (**C**) Enzymatic restriction analysis of pJH222.HIVA^CAT^ plasmid DNA extracted from both the Master Seed (MS, lanes 1–5) and the Working Stock (WS, lanes 7–11) of BCG.HIVA^CAT^ cultures. Lane 1 and lane 7: uncut plasmid; lane 2 and lane 8: HpaI digestion; lane 3 and 9: KpnI digestion; lane 4 and 10: digestion with SpeI; lane 5 and 11: digestion with HindIII; lane 6 and 12: Molecular Weight Marker (1 kb Plus, Invitrogen). (**D**) PCR analysis of HIVA DNA coding sequence using as template the cultures of BCG.HIVA^CAT^ Master Seed (lane 1), and Working Stock (lane 2), Molecular Weight Marker (lane 3), positive control plasmid DNA pJH222.HIVA (lane 4).

In order to confirm that our recombinant BCG.HIVA^CAT^ vaccine strain corresponds to *M. bovis* BCG strain, we used the GenoType MTBC assay based on a commercially available DNA strip assay (Hain Lifescience GmbH, Nehren, Germany) intended for the differentiation of members of the *Mycobacterium tuberculosis* complex (MTBC) and identification of *M. bovis* BCG. This assay is based on *gyrB* DNA sequence polymorphisms and the RD1 deletion of *M. bovis* BCG. Specific oligonucleotides targeting these polymorphisms are immobilized on membrane strips. Amplicons derived from a multiplex PCR react with these probes during hybridization. Each strip has a total of 13 reaction zones. Amplification bands 4–13 include specific probes for each of the tuberculosis complex species. The combination of several hybridization patterns enables to identify the different species of MTBC. Interpretation of the GenoType MTBC hybridization patterns was performed on the basis of the description included in the test. The hybridization patterns were all unequivocal and could easily be allocated to species. Sample results were then compared with the classical differentiation results. We tested four samples corresponding to commercial BCG Connaught, BCG.HIVA^222^, BCG.HIVA^CAT^ (clone10) and BCG Pasteur strains. As we show in Figure 2A, all four strains presented the same hybridization pattern corresponding to *M. bovis* BCG, detecting the bands 4, 7, 9, 10 and 13 belonging specifically to BCG hybridization pattern.

Distribution of BCG to several countries for worldwide application started around 1924 and it was preserved by *in vitro* subculture passaging until 1960s. Since then the Pasteur strain has been freeze-dried, keeping the form of the primary seed lot. The *in vitro* evolution of BCG has resulted in a number of BCG substrains that are heterogenic [34]–[37]. Genetic identification techniques have been used to differentiate diverse BCG substrains: i) the gene probe based on IS986; ii) restriction fragments patterns; iii) whole-genome DNA microarray and iv) multiplex PCR. Using this last method it was reported that the deletion of RD1 occurred in 23 of 23 BCG strains tested. In order to confirm that our BCG.HIVA^CAT^ vaccine strain correspond to BCG Pasteur substrain, we have used the method described by Bedwell *et al.* [38] based on multiplex PCR system targeting SenX3-RegX3 system and the BCG deletion regions including RD1, 2, 8, 14 and 16. Using this method, the BCG vaccine substrains studied could be differentiated into seven fingerprints and all BCG substrains were confirmed. We tested the following samples: BCG.HIVA^CAT^ strain (clone10) Pasteur substrain and commercial BCG Danish 1331 strain. Both BCG substrains evaluated gave a 196 bp product with primers ET1-3, indicating deletion of the RD1 region. In addition in BCG Pasteur (BCG.HIVA^CAT^) the RD8 and RD16 were present and gave a product of 472 and 401 bp respectively. The primers for the SenX3-RegX3 region gave a product of 276 bp in BCG Pasteur. The PCR fingerprints of BCG Pasteur and BCG Danish substrains (Figure 2B) were consistent with previously published results on genetic information of BCG substrains [38]. As shown in Figure 2B, the yield of the PCR was higher when the single primer pairs were used, instead of multiplex format.

For the molecular characterization of pJH222.HIVA^CAT^ plasmid DNA, enzymatic restriction and PCR analysis were performed. The plasmid DNA was isolated from the Master seed and Working stock of BCG.HIVA^CAT^ strain and was characterized. The enzymatic restriction pattern obtained did not show any difference with the predicted enzymatic pattern of the plasmid DNA sequence. HpaI (lanes 2 and 8): band of 6857 bp; KpnI (lanes 3 and 9): bands of 3758, 2117 and 982 bp; SpeI (lanes 4 and 10): band of 6815 bp; HindIII (lanes 5 and 11): bands of 5228 and 1629 bp (Figure 2C). On the other hand, the PCR analysis using specific primers for the HIVA DNA coding sequence was performed using the BCG liquid culture from BCG.HIVA^CAT^ Master seed and Working stock as template. A band of 1776 bp corresponding to HIVA DNA fragment was detected (Figure 2D).

### Phenotypic characterization of the BCG.HIVA^CAT^

**Figure 3:**
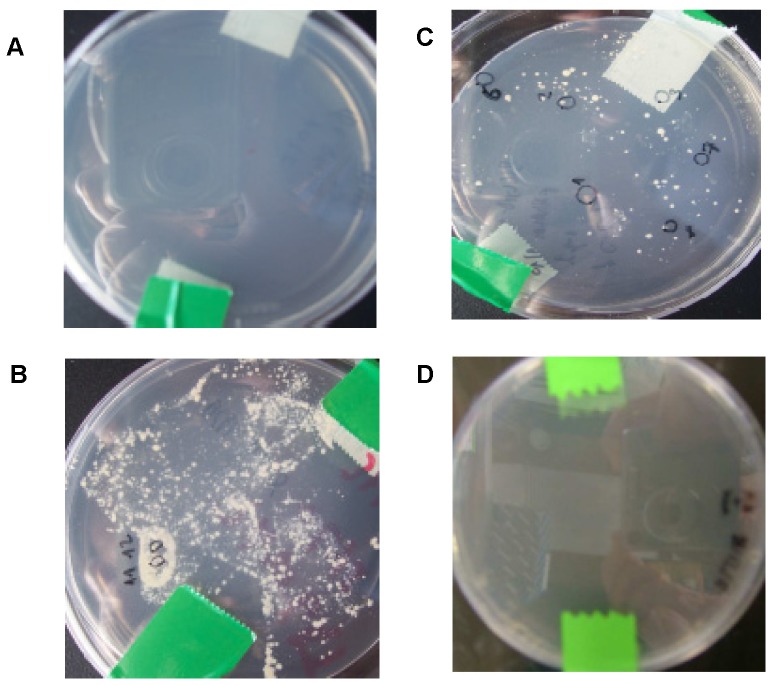
Phenotypic characterization of the BCG.HIVA^CAT^. We assessed the phenotype of lysine auxotrophy, lysine complementation and kanamycin resistance of BCG.HIVA**^CAT^** strain. (**A**) BCG lysine auxotroph strain plated on non-lysine supplemented 7H10; (**B**) BCG lysine auxotroph strain plated on lysine supplemented 7H10; (**C**) BCG.HIVA**^CAT^** plated on 7H10 without lysine and kanamycin supplementation; (**D**) BCG.HIVA^CAT^ plated on 7H10 without lysine supplementation and with kanamycin.

To prevent the plasmid instability *in vivo* and *in vitro* and the genetic rearrangement by mycobacteria, different approaches should be considered: i) the use of expression vectors containing small HIV-1 DNA coding sequences, ii) DNA fragments lacking glycosylation sites; iii) the use of weak promoters; iv) the use of BCG auxotrophic strains (containing the complementing gene in the expression vectors); v) the use of inducible promoters; vi) codon optimization of the recombinant gene; vii) the choice of expression vector backbone and viii) antigen secretion to enhance the immunogenicity and to prevent foreign proteins from becoming toxic to BCG. We have demonstrated that the use of weak promoters (*Mycobacteria spp*. α-antigen promoter) and BCG lysine auxotrophs complemented with a lysine gene do, in fact, prevent the disruption of gene expression caused by genetic rearrangements [23]. In this study, we have used a BCG strain auxotroph for lysine complemented with a lysine gene and antibiotic-free plasmid selection system (no kanamycin resistance). We assessed the phenotype stability of lysine auxotrophy, lysine complementation and kanamycin resistance of BCG.HIVA^CAT^ strain. Initially, the BCG lysine auxotroph strain was plated out on lysine supplemented and non supplemented agar. Such strain failed to grow on non lysine supplemented agar plates and no colonies were observed (Figure 3A). However, growth was observed on agar plates supplemented with lysine (Figure 3B). As expected, complementation of BCG.HIVA^CAT^ strain with lysine gene provided on the multicopy plasmid pJH222.HIVA^CAT^ abolished the requirement for exogenous lysine (Figure 3C). On the other hand, when BCG.HIVA^CAT^ strain was plated out on agar plates containing kanamycin, no colonies were observed (Figure 3D), confirming the lack of kanamycin resistance in our construct.

### BCG.HIVA^CAT^ prime and MVA.HIVA boost regimen elicited HIV-1-specific CD8^+^ and PPD-specific T-cell responses in mice

**Figure 4:**
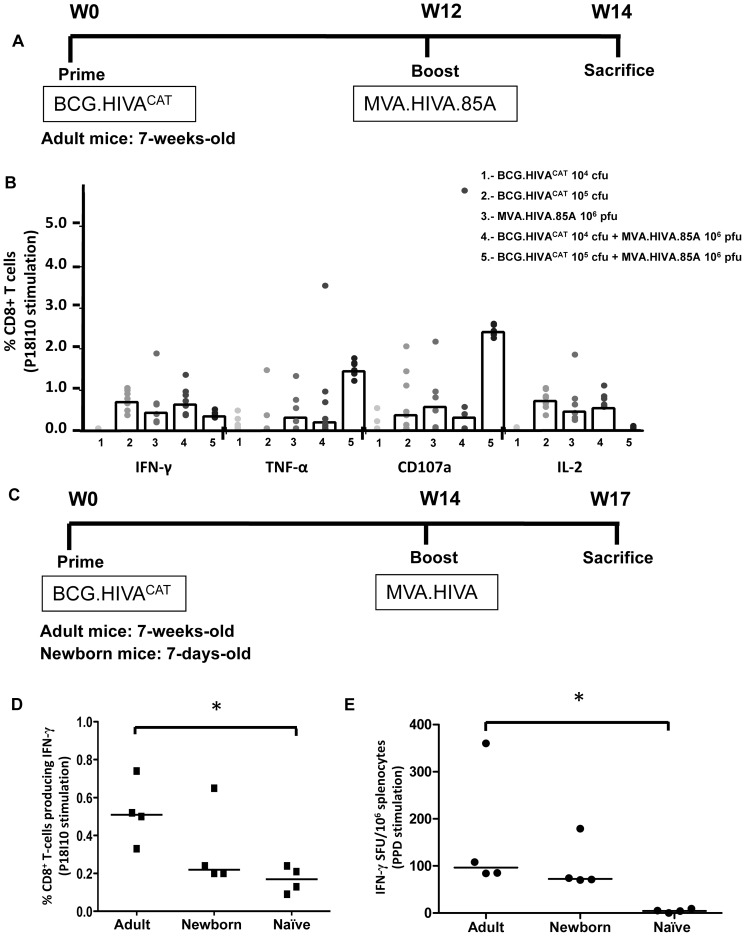
Induction of HIV-1- and *Mtb*-specific T-cells responses by the BCG.HIVA^CAT^ prime - MVA.HIVA boost regimen. (**A**) Adult mice (7-weeks-old) immunized with either 10^4^ or 10^5^ cfu of BCG.HIVA^CAT^ alone (subcutaneously), 10^6^ pfu of MVA.HIVA.85A alone (intramuscularly), or 10^4^ or 10^5^ cfu of BCG.HIVA^CAT^ as a prime and boosted with 10^6^ pfu of MVA.HIVA.85A (left to right). Mice were sacrificed 2 weeks later for T-cell analysis. (**B**) Analysis of IFN-γ, TNF-α, CD107a and IL-2 vaccine elicited HIV-1-specific CD8^+^ T-cell responses. The frequencies of cells producing cytokine are shown. Data are presented as group medians as well as individual animal responses (n = 5). (**C**) Adult and newborn mice (7-days-old) were either left unimmunized or immunized with 2×10^6^ cfu of BCG.HIVA^CAT^ (intradermal and subcutaneous route respectively) and subsequently given a booster dose of 10^6^ pfu of MVA.HIVA (intramuscularly) at 14 weeks post BCG immunization, and sacrificed 3 weeks later. (**D**) Analysis of IFN-γ vaccine elicited HIV-1-specific CD8^+^ T-cell responses. The frequencies of cells producing cytokine are shown. Data are presented as group medians as well as individual animal responses (n = 4). (**E**) PPD-specific T-cell responses elicited by BCG.HIVA^CAT^. Immune responses to BCG were assessed in an *ex vivo* IFN-γ ELISPOT assay using PPD as the antigen. The median spot-forming units (SFU) per 10^6^ splenocytes for each group of mice (n = 4) as well as individual animal responses is shown. * = p<0.05.

We have demonstrated in previous studies in BALB/c mice that BCG.HIVA^222^ can both prime novel and boost preexisting MVA.HIVA elicited HIV-1 specific CD4^+^ and CD8^+^ T-cells immune responses of high quality upon antigenic reexposure. In this study, we have evaluated the specific HIV-1 T-cell immune responses in adult and newborn BALB/c mice after immunization with BCG.HIVA^CAT^ prime and MVA.HIVA or MVA.HIVA.85A boost. The immunogenicity readout was focused on the P18-I10 epitope, an immunodominant CTL epitope derived from HIV-1 Env and H-2D^d^ murine restricted, which was fused to HIVA immunogen to evaluate the immunogenity in mice (Figure 1A). On day 0, adult mice were immunized with rBCG with the episomal plasmid, and on week 12 the animals received a booster dose with MVA.HIVA.85A (Figure 4A). On week 14, the mice were sacrificed and the functional quality of the elicited CD8^+^ T-cells to produce IFN-γ, TNF-α, IL-2 and to degranulate (surface expression of CD107a) in response to P18-I10 peptide stimulation was measured by intracellular cytokine staining (ICS) (Figure 4B). At the higher dose, BCG.HIVA^CAT^ alone and in combination with MVA.HIVA.85A induced HIV-1-specific CD8+ T-cells, producing IFN-γ, TNF-α, and CD107a. For TNF-α, and CD107a, there was a trend of increased responses following MVA.HIVA.85A boost if these were primed by the BCG.HIVA^CAT^ vaccine. In another animal experiment, on day 0, adult and newborn mice were immunized with rBCG, and on week 14 the animals received a booster dose with MVA.HIVA (Figure 4C). We have observed in adult mice that BCG.HIVA^CAT^ prime and MVA.HIVA boost induced higher frequencies of P18-I10 epitope specific CD8^+^ splenocytes producing IFN-γ than newborn and naïve mice (p<0.05) (Figure 4D). These data are in concordance with our previously published results in which the proportions of HIV-1 specific T-cells producing IFN-γ and TNF-α were higher in adult mice compared with newborn mice [24]. Moreover, the magnitude of the bifunctional response was also lower in newborn mice than in adult mice.

BCG.HIVA^CAT^ elicited PPD-specific responses in mice. The BCG-specific immune responses were assessed following the vaccine regimen consisting of BCG.HIVA^CAT^ prime and MVA.HIVA boost as described in Figure 4C. The capacity of splenocytes from vaccinated mice to secrete IFN-γ was tested by ELISPOT assays. The splenocytes secreted IFN-γ after overnight stimulation with the PPD antigen. The frequencies of specific cells secreting IFN-γ was higher in adult mice than in newborn and naïve mice (p<0.05) (Figure 4E).

### BCG.HIVA^CAT^ prime and MVA.HIVA boost was well tolerated in newborn mice

**Figure 5:**
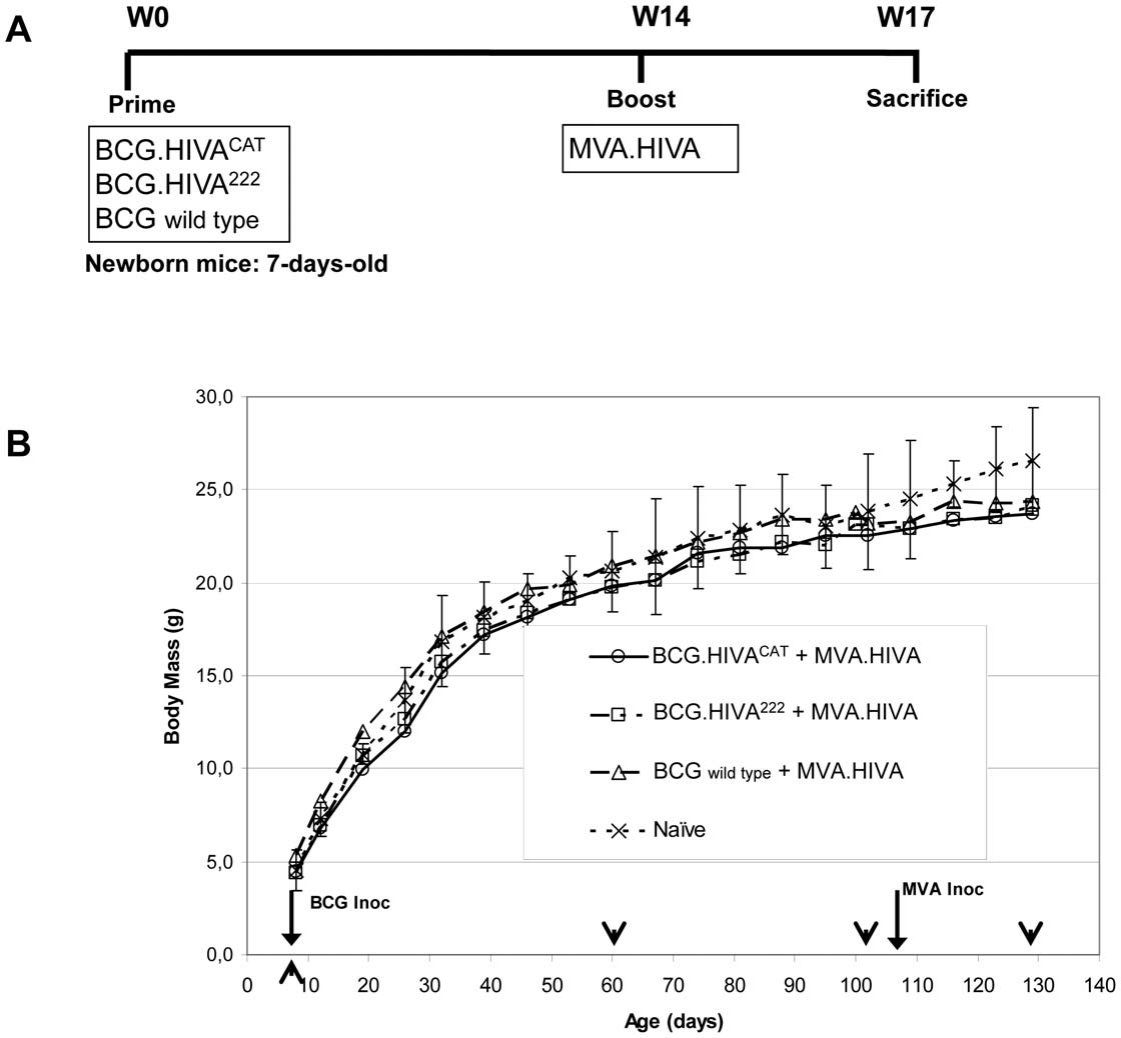
BCG.HIVA^CAT^ prime and MVA.HIVA boost safety in newborn mice. (**A**) Newborn mice were either left unimmunized or immunized with 2×10^6^ cfu of BCG wild type, BCG:HIVA^222^ or BCG.HIVA^CAT^ by subcutaneous route and subsequently given a booster dose of 10^6^ pfu of MVA.HIVA at week 14. (**B**) The body weight was weekly recorded, and the mean for each group of mice is shown (n = 10). Data from naive mice are presented as mean ± 2 SEM (n = 6); At specific time points the weight differences between vaccinated and naïve mice group were analyzed by ANOVA test (arrowheads).

Ten newborn mice (7-days-old) per group were either immunized or left unimmunized with 2×10^6^ colony forming units (cfu) of BCG wild type, BCG.HIVA^222^ or BCG.HIVA^CAT^ via subcutaneous route and subsequently given a booster dose of 10^6^ plaque forming units (pfu) of MVA.HIVA via intramuscular as described in Figure 5A. As shown in Figure 5B, the body mass was weekly monitored and recorded. All vaccine combinations were analyzed, to depict any possible adverse events due to vaccination and monitored by body mass loss. For rigorous safety assessment, the dose inoculated to newborn mice was 10- fold higher, as advised by the European Pharmacopoeia for the safety testing of live vaccines, in comparison with the most usual inoculation dose in adult mice [39]. Importantly, no statistically significant difference was observed between the vaccinated mice groups and the naïve mice group at specific time points, corresponding to BCG inoculation, 2 months after BCG inoculation, pre-MVA boosting and three weeks post MVA-boosting. On the other hand, the body mass profile was similar in all mice groups and similar to mice provider company standard body mass curve (www.Harlan.com). Furthermore, between week 0 and week 14, the body mass monitored in all vaccinated mice groups was found between the mean ± 2 standard deviations (SD) body mass curve in naïve mice (Figure 5B). It is also important to mention that no mice died during the trial, no local adverse events, and no associated systemic reactions were observed.

## Discussion

Despite the progress made on prevention of mother-to-child HIV-1 transmission, the development of a safe, effective and affordable vaccine against HIV-1 and TB at the earliest time after birth to prevent breast milk HIV-1 transmission and childhood tuberculosis is still a great challenge. In this study, i) we have constructed the *E. coli*-mycobacterial shuttle vector to express the HIVA immunogen by using an antibiotic-free plasmid selection system; ii) the genetic and phenotypic characterization of the recombinant lysine auxotroph of BCG.HIVA^CAT^ strain was performed; iii) the HIVA protein expression was confirmed; iv) the specific HIV-1 and *Mtb*-specific immune responses after adult and newborn mice immunization with BCG.HIVA^CAT^ prime and MVA.HIVA or MVA.HIVA.85A boost was evaluated and v) the biosafety profile after newborn mice immunization was monitored. The BCG.HIVA^CAT^ strain was developed in GLP-compatible conditions, preserved by the seed-lot system and was genetically and phenotypically characterized. The *E. coli*-mycobacterial shuttle vector that contains the antibiotic-free plasmid selection system was stable *in vivo* for at least 20 weeks after mice immunization and was used to construct a markerless BCG.HIVA^CAT^ vaccine suitable for Good Manufacturing Practice. Overall, we have demonstrated that BCG.HIVA^CAT^ prime-MVA.HIVA boost regimen was well tolerated in newborn mice and induced HIV-1 and *Mtb*-specific immune responses in adult and newborn mice. Thus, this strategy might be worthy to pursue and for joining the global efforts to develop novel BCG vector-based vaccines for controlling TB and HIV/AIDS.

Even though, it has been described that antibiotics and antibiotic resistance genes have been traditionally used for the selection and maintenance of recombinant plasmids in hosts such as *Escherichia coli*, their use has been considered unacceptable for clinical trials and product licensing. Several approaches have been pursued to replace antibiotics as selective markers for plasmid stability in bacteria, including plasmids harboring gene complementation of a host auxotrophy. In our study, we have used the Operator-Repressor Titration system (ORT) reported by Cranenburgh *et al.* [31], that utilizes *E. coli* DH1*lacdapD* strain that enables plasmid selection and maintenance that is free from antibiotics and selectable marker genes. This is achieved by using only the *lac* operator (*lacO*) sequence as a selectable element. On the other hand the *E. coli*-mycobacterial expression vector contains the lysine A complementing gene of lysine auxotroph of BCG.

Classically, identification of the individual species that comprise *Mycobacterium tuberculosis* complex (MTBC), *M. tuberculosis*, *M. bovis*, *M. bovis* BCG, *M. africanum*, *M. microti*, and *M. canetti*, has been based on phenotypic characteristics and biochemical tests [40]. These tests are slow, need sufficient bacterial growth, are time consuming *and the* interpretation is subjective and could provide identification errors. To overcome these problems, the use of more reliable methods based on molecular biology techniques is necessary. Several DNA-based techniques have been described for the differentiation of members of the MTBC. SpoIigotyping and other molecular methods have been useful tools for rapid species differentiation [41]–[43]. The RD1 deletion identification by PCR was found to be useful for *M. bovis* BCG identification [44]. Niemann *et al.* [45] have established a PCR-restriction fragment length polymorphism assay that allows rapid differentiation of *M. bovis* subsp. *bovis*, *M. bovis* subsp. c*aprae*, and *M. microti*. The same group in 2003 evaluated a commercially available DNA strip assay (Genotype MTBC) for differentiation of clinical MTBC isolates [46]. In this work, we have used also the GenoType MTBC assay to identify our recombinant *M. bovis* BCG vaccine candidate strain. The hybridization pattern obtained was unequivocal and corresponded to *M. bovis* BCG.

The conventional methods for the identification of *Mycobacterium bovis* BCG (BCG) vaccines, based on microscopic examination, biochemical tests and morphological appearance, provided only limited substrain differentiation and no specificity for BCG. The best way to identify different BCG substrains is by using molecular methods and genomic approaches. Differences between BCG substrains have been detected by i) TB genome sequencing [47]; ii) DNA microarray technology [48]; iii) PCR methods [49] and multiplex PCR identity test for BCG vaccines described by Bedwell *et al.* [38]. The PCR fingerprints produced from DNA samples were concordant with predictions based on genetic information on BCG substrains. The capability of this multiplex PCR to discriminate between BCG substrains was tested using commercial preparations and was proven also to be suitable for identification of BCG in clinical samples as well as vaccines. Specific identification of BCG isolates from a variety of clinical situations including immunosuppresed children and adults undergoing therapy for bladder cancer has been performed by using the multiplex PCR based on RD1 deletion region. In this work, we have used the multiplex PCR assay to identify our BCG.HIVA^CAT^ vaccine candidate. Resultant fingerprints after multiplex PCR assay of our BCG vaccine Pasteur substrain, were consistent with the PCR pattern of BCG Pasteur.

Our group and others have shown in murine and non-human primates studies, that rBCG elicited cell-mediated responses against HIV-1 and simian immunodeficiency virus antigens [20], [24], [50]–[52]. However, a small proportion of these animal studies used rBCG strains in heterologous prime-boost regimens. Ami *et al.* [53] have demonstrated that macaques vaccinated with rBCG expressing SIV *gag* and boosted with replication defective poxvirus-SIV *gag*, elicited effective protective immunity against mucosal challenge with SHIV KS661c. There is data showing that rBCG is a good priming vector in heterologous prime-boost vaccination regimens with attenuated virus or recombinant proteins to enhance specific T-cell responses [10], [54], [55]. In tuberculosis vaccine human trials, McShane *et al.* [56], have demonstrated that vaccination with MVA expressing Ag85A boosts pre-existing antimycobacterial immune responses induced either by environmental mycobacteria or BCG vaccination. Hovav *et al*. [57] have shown that priming with recombinant *Mycobacterium smegmatis* expressing HIV-1 gp120 protein induced a cellular immune response that is biased towards memory CD8^+^ T-cells and that can expand dramatically on reexposure to an HIV-1 envelope antigen. We have previously shown in BALB/c mice that the inclusion of BCG.HIVA^222^ in a heterologous prime-boost regimen can prime and increase the HIV-1 specific T-cell immune responses elicited by MVA.HIVA and MVA.HIVA.Ag85A [10], [24], [25], [58]. In addition, BCG.HIVA prime and MVA.HIVA boost-elicited HIV-1 specific CD8^+^ T-cells exhibited effector functions such as production of IFN-γ and TNF-α. These HIV-1-specific T-cell responses were higher in adult than in newborn mice. The prime-boost regimen consistently enhanced and improved the frequency, quality and durability of the generated HIV-1 specific responses in adult and newborn mice. This improvement was observed by the detection of the highest bifunctional HIVA-specific T-cell responses and higher specific cytolytic activity in the mice that received BCG.HIVA versus BCG wild type. These data are consistent with the specific HIV-1 specific immune responses detected in this study after newborn and adult mice immunization with BCG.HIVA^CAT^ prime and MVA.HIVA boost, observing higher HIV-1 specific T-cell responses in adult than in newborn mice. In addition, in adult mice, BCG.HIVA^CAT^ primed and enhanced the MVA.HIVA.85A-elicited HIV-1-specific CD8^+^ T-cell responses. There are few reports in the literature describing the safety and immunogenicity of rBCG expressing HIV-1 antigens in neonatal mice and neonatal non-human primates. Ranganathan *et al*. [9] have evaluated the immunogenicity in neonatal mice of three different recombinant attenuated *Mtb* strains expressing an HIV-1 envelope and showed that single dose immunization in neonatal mice with ΔlysA ΔsecA2 *Mtb* strain expressing HIV-1 Env rapidly generated HIV-1 and *Mtb-* specific T-cell immune responses. In the present study, we showed in newborn mice that BCG.HIVA^CAT^ prime and MVA.HIVA boost increased the frequencies of specific CD8^+^ T-cells producing IFN-γ. We observed in newborn mice a lower level of HIV-1 specific T-cell immune responses compared with adult mice. Rosario *et al.* [59] have assessed the immunogenicity of the BCG.HIVA^222^ prime and MVA.HIVA boost regimen in newborn rhesus macaques and made similar observation. On the other hand, we suggest that additional experiments should be performed in newborn mice inoculating the rBCG expressing HIV-1 antigens by different routes and different doses, because the route and dose of neonatal vaccination may provide different levels of immune activation, which may affect the efficacy of the vaccine.

Here, the vaccination with BCG.HIVA^CAT^ strain induced BCG-specific responses in adult and newborn mice. Studies in neonatal mice have indicated that immune responses at birth are often biased towards the Th2 type and defective in the Th1 type, the central defense mechanism against intracellular pathogens. However, it has been described that BCG vaccination at birth induces a potent Th1-type immune response in humans and in mice [60], [61].

The challenge for neonatal vaccinology is thus to develop, and promote at a global level, vaccines that could be safely administered soon after birth and would be effective after one or two early doses. According to our knowledge, no reports have been published about safety of antibiotic-free marker recombinant BCG based HIV-1 vaccine in neonatal mice. Rosario *et al.* [59] have demonstrated that BCG.HIVA^401^ followed by two doses of MVA.HIVA in rhesus macaques was safe, not associated with systemic reactions and the local adverse events detected were considered to be consistent with a predicted response to the BCG vaccine administration, similar to that observed in human neonates. In the present study, we have demonstrated in neonatal mice (7-days-old) by the rate of body mass that BCG.HIVA^CAT^ prime and MVA.HIVA boost regimen was well tolerated.

In conclusion, we constructed and characterized a novel, safer, GLP-compatible BCG-vectored vaccine using prototype immunogen HIVA and tested the safety and immunogenicity of BCG.HIVA^CAT^and MVA.HIVA in newborn and adult mice using the prime-boost regimen. BCG expressing a second generation immunogen HIVconsv better addressing the HIV-1 variability and escape [62] is under construction. The same strategy can be easily used for other major pediatric pathogens.

## Materials and Methods

### Construction of BCG.HIVA^CAT^ strain by using an antibiotic-free plasmid selection system and expressing HIV-1 clade A immunogen

Parental *E. coli-*mycobacterial shuttle vector, plasmid pJH222, was kindly provided by W. R. Jacobs Jr., B.R. Bloom, and T. Hsu. The coding sequence of the HIVA gene (derived from consensus HIV-1 clade A Gag protein, an immunogen derived from an HIV-1 strain prevalent in central and eastern Africa, and a string of CD8^+^ T-cell epitopes) was fused to the *M. tuberculosis* nucleotides coding for the 19-kDa lipoprotein signal sequence by PCR, and the chimeric gene was cloned into the pJH222 as a HindIII-HindIII fragment under the control of the *M. tuberculosis* α-antigen promoter by using standard recombinant-DNA techniques. Plasmid DNA pJH222 is a replicative (multicopy, extrachromosomal) vector that contains a DNA cassette encoding kanamycin resistance (Tn903-derived *aph* gene), an *E. coli* origin of replication (*oriE*), and a mycobacterial plasmid DNA origin of replication (*oriM*). It contains also the wild-type lysine A-complementing gene for the vector maintenance (*lysA5*) in the BCG lysine auxotroph [4]. The aminoglycoside phosphotransferase gene (aph), conferring kanamycin resistance, was removed from pJH222.HIVA plasmid by using the Operator-Repressor Titration (ORT) system, developed by Cobra Biologics, Oxford, UK [30]. Following *aph* gene excision by plasmid DNA SpeI digestion, a tighter-binding 20 bp variant of the *lacO* sequence (generated by annealing complementary oligonucleotides) was ligated and transformed into *E. coli* DH1*lacdapD* strain.

### Mycobacterial strains and culture

The pJH222.HIVA plasmid DNA without kanamycin resistance gene, here designated as pJH222.HIVA^CAT^ plasmid, was transformed by electroporation in a lysine auxotroph of BCG, kindly provided by W.R. Jacobs Jr., B.R. Bloom, and T. Hsu. Mycobacterial cultures were grown in Middlebrook 7H9 broth medium or on Middlebrook agar 7H10 medium supplemented with albumin-dextrose-catalase (ADC; Difco) and containing 0.05% Tween 80. The L-lysine monohydrochloride (Sigma) was dissolved in distilled water and used at a concentration of 40 µg/ml. For transformation, BCG cultures were grown to an optical density at 600 nm of 0.9, transformed using a Bio-Rad gene pulser electroporator at 2.5 kV, 25 mF, and 1,000 Ω, and plated onto ADC-supplemented Middlebrook agar 7H10 medium containing 0.05% Tween 80. Commercial BCG Danish 1331 strain (Pfizer), was kindly provided by Dr. Neus Altet and commercial BCG Connaught strain (ImmuCyst, Aventis), from the Urology Department at Hospital Clínic de Barcelona.

### Dot-blot analysis

Cell lysates of mid-logarithmic-phase BCG transformants were prepared by sonication and using a protein extraction buffer (50 mM Tris-HCl pH 7.5, 5 mM EDTA, 0.6% sodium dodecyl sulphate) and 100× protease inhibitor cocktail (1 mg/ml aprotinin, 1 mg/ml E-64, 1 mg/ml leupeptin, 1 mg/ml pepstatin A, 50 mg/ml pefabloc SC, and 10 ml dimethyl sulfoxide). The protein extract was blotted onto a pre-treated PVDF membrane, and HIVA protein was detected using anti-Pk monoclonal antibodies (MCA1360 AbD Serotec), with an ECL kit (Pierce). To visualize the dots, the Typhoon 8600 gel imaging system (GE Healthcare) was used.

### *In vivo* stability of plasmid pJH222.HIVA^CAT^

The growth of rBCG and the *in vivo* stability of the extrachromosomal plasmid pJH222.HIVA^CAT^ were established by the recovery of BCG.HIVA^CAT^ colonies from the spleens after 20 weeks after mice immunization with 10^5^ cfu of BCG.HIVA^CAT^. Spleens were homogenized and plated onto Middlebrook 7H10 medium supplemented with ADC (Difco) and containing 0.05% Tween 80. The resulting colonies were inoculated in 7H9 medium supplemented with ADC and 0.05% Tween 80. The DNA coding sequence corresponding to HIVA immunogen was detected by PCR analysis, using the BCG liquid culture as a template.

### Sample preparation for the GenoType MTBC assay and Multiplex PCR assay

For isolation of DNA from BCGwt, BCG.HIVA^222^, and BCG.HIVA^CAT^strains, 400 µl of mycobacterial culture were centrifuged at 13000×g for 10 minutes, at room temperature, the pellet was resuspended in 250 µl of distilled water, and heated to 95°C in a thermoblock for 15 minutes to lyse and inactivate vegetative bacterial forms. Finally, after 5 minutes centrifugation at 10,000×g, 5 µl of supernatant were used for the amplification reaction or stored at −20°C. The commercial BCG strains were treated in a similar way, but in this case, 400 µl of the reconstituted freeze-dried flask were used.

### GenoType MTBC assay for *M. bovis* BCG identification

The Mycobacterium bovis BCG strain identification was performed with a commercially available system based on DNA hybridization technology on nitrocellulose strips (GenoType MTBC; Hain Diagnostika, Nehren, Germany). The GenoType MTBC assay is based on an *M. tuberculosis* complex-specific 23S ribosomal DNA fragment, *gyrB* DNA sequence polymorphisms, and the RD1 deletion of *M. bovis* BCG. Specific oligonucleotides targeting these polymorphisms are immobilized on membrane strips. Amplicons derived from a multiplex PCR (performed using the biotynilated primers provided with the kit) react with these probes during hybridization. The Genotype MTBC assay was performed as recommended by the manufacturer. Briefly, for amplification, 35 µl of a primer nucleotide mixture (provided with the kit), amplification buffer containing 2.5 mM MgCl_2_ and 1.25 U of HotStarTaq polymerase (Qiagen, Hilden, Germany), and 5 µl of DNA (see above sample preparation paragraph) in a final volume of 50 µl were used. The amplification protocol consisted of 15 min of denaturation at 95°C, followed by 10 cycles comprising 30 s at 95°C and 120 s at 58°C, an additional 20 cycles comprising 25 s at 95°C, 40 s at 53°C, and 40 s at 70°C, and a final extension at 70°C for 8 min [41]. Each biotin-labeled PCR product was denatured and hybridized to a strip with 13 specific oligonucleotide probes, using a heat-controlled washing and shaking automaton (GT-Blot 48; Hain Lifescience GmbH, Nehren, Germany). The specificity and targeted genes (in parentheses) of the probes were as follows: 1, conjugate (hybridization) control; 2, *Mycobacterium* genus-specific amplification control (23S rRNA); 3, *M. tuberculosis* complex-specific probe for identification control (23S rRNA); 4 to 12, discriminative for *M. tuberculosis* complex species (*gyrB*), and 13, *M. bovis* BCG specific probe (RD1). Six different patterns could be obtained (*M. tuberculosis* or *M. canettii*, *M. africanum*, *M. bovis*, *M. bovis* BCG, *M. caprae*, and *M. microti*). A template sheet showing the positions of the lines and the interpretation table, both provided with the kit, were used for interpretation of the test results.

### Multiplex PCR assay for M. bovis BCG Substrain Pasteur Identification

The multiplex PCR was performed using 13 primers [38] targeting the SenX3–RegX3 system (C3 and C5) and the BCG deletion regions including RD1 (ET1-3), RD2, RD8, RD14 and RD16 regions. For the PCR analysis, 5 µl of the DNA (see above sample preparation paragraph) isolated from BCG.HIVA^CAT^ Pasteur strain and BCG Danish 1331 strain was used in a final volume of 50 µl with the following amplification protocol : 1 cycle at 94°C (10 min) and 30 cycles at 94°C (1 min), 55°C (1 min) and 72°C (2 min), and 1 cycle at 72°C (10 min). The PCR products were analyzed in a 3% (w/v) agarose gel electrophoresis. The PCR fingerprints of BCG Pasteur and BCG Danish substrains were consistent with previously published results on genetic information of BCG substrains [38].

### Mycobacterial plasmid DNA extraction

The rBCG broth culture up to an OD of 0.9 (600 nm) from Master Seed and Working Stock, was used for mycobacterial plasmid DNA isolation. The QIAprep Spin Miniprep Kit (Qiagen, Hilden, Germany) was used with slight modifications :i) prior to harvest (3 to 24 hours), glycine at a final concentration of 1% (w/v) was added; ii) the cell pellet was treated with the P1 buffer from the Miniprep Qiagen kit, supplemented with 10 mg/ml of lysozyme (Sigma) and incubated at 37°C overnight; iii) the extraction column was treated with a 10 ml mixture of chloroform∶isopropanol 1∶1. The mycobacterial plasmid DNA isolated was used for restriction enzyme analysis.

### Mice immunizations and isolation of splenocytes

Adult (7-weeks-old) and newborn (7-days-old) female BALB/c mice were immunized with BCG.HIVA^CAT^, and were boosted with MVA.HIVA or MVA.HIVA.85A at doses, routes and schedules outlined in the figure legends. On the day of sacrifice, individual spleens were collected and splenocytes were isolated by pressing spleens through a cell strainer (Falcon) using a 5-ml syringe rubber plunger. Following the removal of red blood cells with ACK lysing buffer (Lonza), the splenocytes were washed and resuspended in complete medium (R10 [RPMI 1640 supplemented with 10% fetal calf serum and penicillin-streptomycin], 20 mM HEPES, and 15 mM 2-mercaptoethanol).

### Ethics statement

The animal experiments were approved by the local Research Ethics Committee (Clinical Medicine, School of Medicine, University of Barcelona and University of Oxford) and by the Ethical Committee for animal experimentation from University of Barcelona and University of Oxford. All animal procedures and care conformed strictly to Catalonia (Spain) and to United Kingdom Animal Welfare legislation.

### Ex Vivo IFN-γ ELISPOT Assay

The ELISPOT assay was performed using a commercial IFN-*γ* ELISPOT kit (Mabtech, Sweden). The ELISPOT plates (Millipore, MSISP4510, 96 wells plates with PVDF membranes) were coated with purified antimouse IFN-*γ* capture monoclonal antibody diluted in PBS to a final concentration of 5 µg/ml at 4°C overnight. The plates were washed once in R10 and blocked for 2 h with R10. A total of 5×10^5^ fresh splenocytes were added to each well, stimulated with 5 µg/ml of PPD for 16 h at 37°C, 5% CO_2_, and lysed by incubating twice with deionized water for 5 minutes. Wells were then washed 3× with PBS 0.05% Tween 20, incubated for 2 h with a biotinylated anti-IFN-*γ* mAb diluted in PBS 2% FCS to a final concentration of 2 ug/ml, washed 3× in PBS 0.05% Tween 20, and incubated with the Streptavidin-Alkaline Phosphatase-conjugate in PBS 2% FCS. Wells were washed 4× with PBS 0.05% Tween 20 and 2× with PBS before incubating with 100 µl BCIP/NBT substrate solution (Sigma). After 5–10 minutes, the plates were washed with tap water, dried, and the resulting spots counted using an ELISPOT reader (Autoimmune Diagnostika GmbH, Germany).

### Intracellular Cytokine Staining

One million splenocytes were added to each well of a 96-well round-bottomed plate (Costar) and pulsed with 2 µg/ml of P18-I10 peptide (RGPGRAFVTI) [23] and kept at 37°C, 5% CO_2_ for 60 minutes, followed by the addition of GolgiStop (BD Biosciences) containing monensin. After a further 5-hour incubation, reaction was terminated by storing the plate at 4°C. The cells were washed with FACS wash buffer (PBS, 2% FCS, 0.01% Azide) and blocked with anti-CD16/32 (BD Biosciences) at 4°C for 30 minutes. All subsequent antibody stains were performed using the same conditions. Cells were then washed and stained with anti-CD8-PerCP (BD Biosciences) and anti CD107a-FITC, washed again, and permeabilized using the Cytofix/Cytoperm kit (BD Biosciences). Perm/Wash buffer (BD Biosciences) was used to wash cells before staining with anti-IFN-γ-APC, anti TNF-α-PE and anti IL-2 Alexa Fluor 647 (BD Biosciences). Cells were fixed with CellFIX (BD) and stored at 4°C until analysis.

### Fluorescence-Activated Cell Sorter Analysis

All chromogen-labeled cells were analyzed in a Becton Dickinson FACScalibur, using the CellQuest software for acquisition (BD Biosciences) and the Flow-Jo software (Tri-Star) for analysis.

### Statistical analysis

Immunogenicity data are shown as group medians as well as individual responses. The body mass data are group means, and mean ± 2 SD in naïve mice group. Statistical significance was determined by ANOVA (* = p<0.05).
